# X-linked congenital adrenal hypoplasia associated with hypospadias in an Egyptian baby: a case report

**DOI:** 10.1186/1752-1947-6-428

**Published:** 2012-12-28

**Authors:** Kotb Abbass Metwalley, Hekma Saad Farghaly

**Affiliations:** 1 Pediatric Endocrinology Unit, Department of Pediatrics, Faculty of Medicine, Assiut University, Assiut, Egypt

## Abstract

**Introduction:**

X-linked congenital adrenal hypoplasia is a rare developmental disorder of the human adrenal cortex and is caused by deletion or mutation of the *dosage-sensitive sex reversal adrenal hypoplasia congenita critical region of the X chromosome, gene 1* (*DAX-1*) gene. Most affected children present with failure to thrive, salt wasting and hypoglycemic convulsions in the first months of life. Hypospadias affects approximately one in 250 live male births. Mutations in the *mastermind-like domain-containing 1* (*MAMLD1*) gene have been implicated as one of the causes of hypospadias in children. To the best of our knowledge, an association between congenital adrenal hypoplasia due to a *DAX-1* mutation and hypospadias due to mutation of the *MAMLD1* gene has not previously been reported in the literature.

**Case presentation:**

A 35-day-old male Egyptian baby was referred to our institution for the evaluation of a two-week history of recurrent vomiting associated with electrolyte imbalance. On examination, our patient was found to have hypotension and dehydration. A genital examination showed distal penile hypospadias with chordee and normal testes. He had hyponatremia, hyperkalemia, hypoglycemia and metabolic acidosis. Endocrinological investigations revealed low levels of cortisol, 17-hydroxyprogesterone and aldosterone, with a high level of adrenocorticotrophic hormone. A provisional diagnosis of congenital adrenal hypoplasia associated with hypospadias was made. A molecular genetics study confirmed the diagnosis of X-linked congenital adrenal hypoplasia due to *DAX-1* mutations and hypospadias due to *MAMLD1* mutation. He was started on hydrocortisone and fludrocortisone treatment. After three weeks of treatment, his symptoms improved and his blood sugar, sodium, potassium and cortisol levels normalized.

**Conclusions:**

We report the case of an Egyptian baby with an association of congenital adrenal hypoplasia due to *DAX-1* mutation and hypospadias due to *MAMLD1* mutation. Early diagnosis of this association and determining its optimal treatment are vital in helping to avoid its fatal course.

## Introduction

Congenital adrenal hypoplasia is a rare hereditary disorder, first described in 1948 by Sikl [1]. Most affected children present with failure to thrive, salt wasting and hypoglycemic convulsions in the first months of life [2]. The primary forms of congenital adrenal hypoplasia appear as X-linked and autosomal recessive disorders with different adrenal morphologies. The X-linked form (OMIM:300200; http://www.ncbi.nlm.nih.gov/omim) is caused by a mutation or deletion of the gene *dosage-sensitive sex reversal adrenal hypoplasia congenita critical region of the X chromosome, gene 1* (*DAX-1*; also called the *adrenal hypoplasia, congenital homolog* (*AHCH*) gene) on the X chromosome [3]. Hypospadias is the displacement of the urethral meatus from the tip of the glans to the ventral side of the phallus. It is one of the most common congenital anomalies and is estimated to occur in between 0.3 and 8.0 of 1000 male births. Most cases have an unknown etiology, which is probably a mix of monogenic and multifactorial forms, implicating both genetic and environmental factors [4]. One responsible mechanism is thought to be incomplete masculinization of the external genitalia. For example, subnormal Leydig cell function in utero, a mild degree of androgen resistance, a 5-α-reductase 2 deficiency, or abnormality in the action of dihydrotestosterone (DHT) can cause hypospadias [5]. In addition, several genes, such as *SRY type HMG box9* (*SOX9)*, *HomeoboxA13 *(*HOXA13)*, *Wilms tumor 1 *(*WT1)* and *mastermind-like domain-containing 1* (*MAMLD1*) [6] are reported to be associated with hypospadias. We present the case of a 35-day-old male Egyptian baby who presented with X-linked congenital adrenal hypoplasia due to a *DAX-1* mutation associated with hypospadias due to a mutation of *MAMLD1*.

## Case presentation

A 35-day-old male Egyptian baby was referred to our institution from a regional hospital for evaluation of a two-week history of recurrent vomiting associated with hyponatremia, hyperkalemia and failure to gain weight. He was the first child of related Egyptian parents. The pregnancy was uneventful and his mother had not been treated with any drugs. The existence of other children with this condition had not previously been mentioned. He was delivered at term by Caesarean section with a birth weight of 3.4kg, and cried immediately after delivery. He required admission to the special care baby unit shortly after birth for hypoglycemia; his blood sugar levels were easily maintained on 10% dextrose and then on milk feeds.

**Figure 1:**
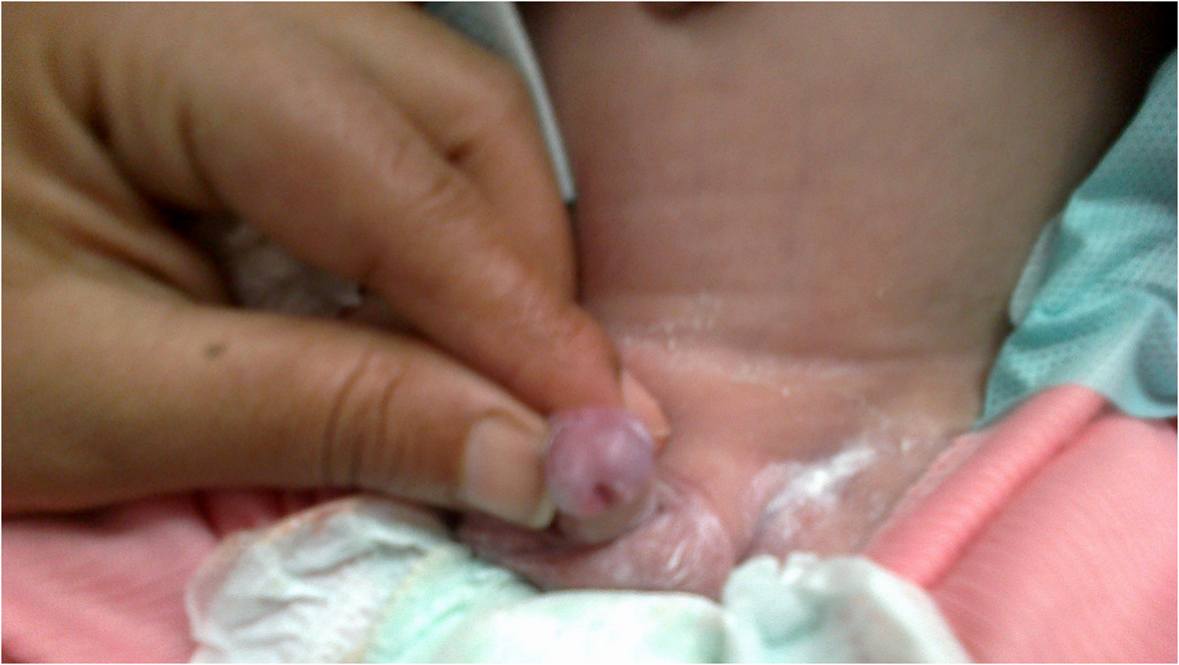
Penile hypospadias in a male Egyptian baby with congenital adrenal hypoplasia.

On examination, our patient’s weight was 3.5kg (below the third percentile), he was dehydrated, his blood pressure was 70/40mmHg, and he had no hyperpigmentation or dysmorphic features. A genital examination revealed distal penile hypospadias with chordee and normal testes (Figure 1). Laboratory investigation results showed a serum sodium level of 119mmol/L, potassium 6.5mmol/L and glucose 55mg/dL. Blood gas analysis results showed a pH of 7.31, bicarbonate ion level of 16.9mmHg and a base excess of 6.3mmol/L. His cortisol level was 0.3μg/dL (normal range: 2.8 to 23μg/dL) and adrenocorticotropic hormone level, 512pg/mL (normal range: 6 to 48pg/mL). An adrenocorticotropic hormone stimulation test did not cause a rise in his cortisol level. His 17-hydroxyprogesterone level was 0.005ng/mL (normal range: 0.03 to 0.9ng/mL), androstenedione 0.012ng/mL (normal range: 0.1 to 0.17ng/mL), dehydroepiandrosterone 6ng/mL (normal range: 50 to 480ng/mL), plasma renin activity 90ng/mL/hour (normal range: 2.35 to 37ng/mL/hour) and aldosterone 31pg/mL (normal range: 50 to 900pg/mL). His triglyceride and creatine kinase levels were normal. His blood karyotype was 46,XY. A magnetic resonance imaging study of his abdomen revealed small adrenal glands and a normal genitourinary system.

Based on the clinical history and investigative findings, a provisional diagnosis of congenital adrenal hypoplasia associated with hypospadias was made. A molecular genetic study detected hemizygous mutations (p.Arg327Pro). A deoxyribonucleic acid (DNA) study of our patient’s mother confirmed her to be heterozygous for the same mutation. No mutation was found in his father. With the identification of a hemizygous mutation in the *DAX-1* gene, a clinical diagnosis of X-linked congenital adrenal hypoplasia was confirmed. Further, mutation c.325delG in the *MAMLD1* gene was detected, which may point to a possible etiology of hypospadias in our patient’s case. He was started on hydrocortisone at a dose of 10mg/m^2^/day and fludrocortisone 0.05mg/day. After three weeks of treatment the vomiting stopped, his weight was 4.1kg and his blood sugar, sodium, potassium and cortisol levels normalized. At the age of three months, a human chorionic gonadotropin (hCG) loading test was performed to evaluate his gonadal function. Basal values of luteinizing hormone and follicle-stimulating hormone were <0.5IU/L and 0.41IU/L, respectively, which is within normal prepubertal limits. The hCG loading test resulted in a normal testosterone response from <0.05ng/mL to 4.24ng/mL. The ratio of testosterone to DHT after hCG loading was 7.3:1.

## Discussion

X-linked congenital adrenal hypoplasia is a rare disease with a prevalence rate of approximately one in 12,500 in the USA [7]; its prevalence in Egypt is unknown. *DAX-1* is expressed in the adrenal cortex, gonads, hypothalamus and anterior pituitary and is essential for the normal development of the steroidogenic axis and sex determination. Over 80 mutations of varying types have been identified in this gene [8]. Patients with a mutation of the *DAX-1* gene usually present with primary adrenal insufficiency in early infancy or childhood, and hypogonadotropic hypogonadism during puberty [9].

Chromosome X-linked heredity is usually recessive in women. In our case, our patient’s mother was heterozygous for the mutation and was therefore a carrier, because the normal dominant allele prevented expression of the affected gene. Her XY sons were hemizygous for the mutation and were therefore affected by the condition [10]. There is phenotypic heterogeneity associated with *DAX-1* mutations. The lack of genotype-phenotype correlation in some mutations is presumably caused by the influence of other modifying genes, which lead to significant within-family variations in age at onset and expression. Adrenal failure may be transient, asymptomatic or manifest in adulthood. However, in the absence of adrenal insufficiency, *DAX-1* mutations are an uncommon cause of hypogonadotropic hypogonadism or pubertal delay [11]. The X-linked type of mutation can be associated with Duchenne muscular dystrophy and/or glycerol kinase deficiency as part of a contiguous gene syndrome. Other unusual presentations, such as progressive high-frequency hearing loss, profound hyperpigmentation, and monosomy have also been described [12].

A clinical diagnosis of congenital adrenal hypoplasia due to *DAX-1* mutations is not always easily made. Boys who present in the neonatal period with salt wasting and adrenal insufficiency are sometimes misdiagnosed with the more common disorder, 21-hydroxylase deficiency (congenital adrenal hyperplasia), although the adrenal steroid profiles of these conditions are quite different. In congenital adrenal hypoplasia, 17-hydroxyprogesterone levels are low, whereas they are increased in congenital adrenal hyperplasia. Distinguishing these two disorders is important because they differ in their clinical course, steroid management and genetic counseling. The recessive form of congenital adrenal hypoplasia should also be considered as a cause of primary adrenal insufficiency in infancy. It has a distinct miniature adult adrenal morphology, characterized by small glands with a permanent cortical zone but a diminished fetal zone. The genetic basis of the recessive form of congenital adrenal hypoplasia is unknown [13].

Normal penile and urethral development begins in the sixth week of gestation with the formation of the urogenital sinus, which eventually becomes masculinized under the direction of testosterone and its more potent form, dihydrotestosterone. Without the presence of adequate levels of testosterone or a functioning androgen receptor, the genital structures become female in appearance, as seen in the most severe cases of hypospadias [14]. Our patient had penile hypospadias without cryptorchidism or genital malformations. Further, his basal values of luteinizing hormone and follicle-stimulating hormone were appropriate for his age, which suggested normal hypophyseal function. An hCG loading test confirmed intact testosterone biosynthesis in his testes. The testosterone/DHT ratio after hCG loading, a test reported to be useful in the evaluation of 5-α-reductase-2 activity was within the normal range at 7.3:1 [15]. These data imply that his androgen biosynthetic pathway was normal. The overwhelming majority of cases of hypospadias remain unexplained, particularly the milder forms. Genes have been implicated as a possible etiology for hypospadias. One of the candidate genes identified for the development of the male genitalia is *MAMLD1*. The mechanism by which *MAMLD1* mutations induce hypospadias remains to be elucidated [16]. Kalfa *et al*. [17] documented that the occurrence of an early nonsense codon in *MAMLD1* is frequently associated with hypospadias.

Hypospadias occurs as an isolated defect or, less commonly, as a feature of numerous genetic syndromes. The isolated form is usually sporadic, however, familial occurrence has been reported. Hypospadias, which is often of variable degree, is not a consistent or pathognomonic feature in syndromic conditions [18].

## Conclusions

We report the case of a male Egyptian baby with an association of congenital adrenal hypoplasia due to a *DAX-1* mutation and hypospadias due to mutation of the *MAMLD1* gene. Early diagnosis of this association and determining its optimal treatment are very important in helping to avoid its fatal course.

## Consent

Written informed consent was obtained from the patient’s next-of-kin for publication of this case report and any accompanying images. A copy of the written consent is available for review by the Editor-in-Chief of this journal.

## Competing interests

The authors declare that they have no competing interests.

## Authors’ contributions

KAM and HSF diagnosed, investigated, followed-up and managed the patient, and drafted the manuscript. Both authors read and approved the final manuscript.
